# A roadmap for the generation of benchmarking resources for antimicrobial resistance detection using next generation sequencing

**DOI:** 10.12688/f1000research.39214.1

**Published:** 2021-02-08

**Authors:** Mauro Petrillo, Marco Fabbri, Dafni Maria Kagkli, Maddalena Querci, Guy Van den Eede, Erik Alm, Derya Aytan-Aktug, Salvador Capella-Gutierrez, Catherine Carrillo, Alessandro Cestaro, Kok-Gan Chan, Teresa Coque, Christoph Endrullat, Ivo Gut, Paul Hammer, Gemma L. Kay, Jean-Yves Madec, Alison E. Mather, Alice Carolyn McHardy, Thierry Naas, Valentina Paracchini, Silke Peter, Arthur Pightling, Barbara Raffael, John Rossen, Etienne Ruppé, Robert Schlaberg, Kevin Vanneste, Lukas M. Weber, Henrik Westh, Alexandre Angers-Loustau

**Affiliations:** 1 European Commission Joint Research Centre, Ispra, Italy; 2 European Commission Joint Research Centre, Geel, Belgium; 3 The European Centre for Disease Prevention and Control, Stockholm, Sweden; 4 National Food Institute, Technical University of Denmark, Lyngby, Denmark; 5 Barcelona Supercomputing Centre (BSC), Barcelona, Spain; 6 Ottawa Laboratory – Carling, Canadian Food Inspection Agency, Ottawa, Ontario, Canada; 7 Fondazione Edmund Mach, San Michele all'Adige (TN), Italy; 8 International Genome Centre, Jiangsu University, Zhenjiang, China; 9 Division of Genetics and Molecular Biology, Institute of Biological Sciences, Faculty of Science, University of Malaya, Kuala Lumpur, Malaysia; 10 Servicio de Microbiología, Hospital Universitario Ramón y Cajal, Instituto Ramón y Cajal de Investigación Sanitaria (IRYCIS), Madrid, Spain; 11 Spanish Consortium for Research on Epidemiology and Public Health (CIBERESP), Carlos III Health Institute, Madrid, Spain; 12 MSD SHARP & DOHME GMBH, Haar, Germany; 13 Centro Nacional de Análisis Genómico, Centre for Genomic Regulation (CNAG-CRG), Barcelona Institute of Technology, Barcelona, Spain; 14 Universitat Pompeu Fabra, Barcelona, Spain; 15 BIOMES. NGS GmbH c/o Technische Hochschule Wildau, Wildau, Germany; 16 Quadram Institute Bioscience, Norwich Research Park, Norwich, UK; 17 Unité Antibiorésistance et Virulence Bactériennes, ANSES Site de Lyon, Lyon, France; 18 University of East Anglia, Norwich, UK; 19 Helmholtz Centre for Infection Research, Braunschweig, Germany; 20 French-NRC for CPEs, Service de Bactériologie-Hygiène, Hôpital de Bicêtre, Le Kremlin-Bicêtre, France; 21 Institute of Medical Microbiology and Hygiene, University of Tübingen, Tübingen, Germany; 22 Center for Food Safety and Applied Nutrition, US Food and Drug Administration, College Park, MD, USA; 23 Department of Medical Microbiology, University Medical Center Groningen, University of Groningen, Groningen, The Netherlands; 24 IAME, Université de Paris, Paris, France; 25 Department of Pathology, University of Utah, Salt Lake City, UT, USA; 26 Transversal activities in Applied Genomics, Sciensano, Brussels, Belgium; 27 Institute of Molecular Life Sciences, University of Zurich, Zurich, Switzerland; 28 SIB Swiss Institute of Bioinformatics, University of Zurich, Zurich, Switzerland; 29 Present address: Department of Biostatistics, Johns Hopkins Bloomberg School of Public Health, Baltimore, MD, USA; 30 Hvidovre University Hospital, Hvidovre, Denmark; 31 European Commission Publications Office, Luxembourg, Luxembourg

**Keywords:** Antimicrobial resistance, bioinformatics, next-generation sequencing, benchmarking

## Abstract

Next Generation Sequencing technologies significantly impact the field of Antimicrobial Resistance (AMR) detection and monitoring, with immediate uses in diagnosis and risk assessment. For this application and in general, considerable challenges remain in demonstrating sufficient trust to act upon the meaningful information produced from raw data, partly because of the reliance on bioinformatics pipelines, which can produce different results and therefore lead to different interpretations. With the constant evolution of the field, it is difficult to identify, harmonise and recommend specific methods for large-scale implementations over time. In this article, we propose to address this challenge through establishing a transparent, performance-based, evaluation approach to provide flexibility in the bioinformatics tools of choice, while demonstrating proficiency in meeting common performance standards. The approach is two-fold: first, a community-driven effort to establish and maintain “live” (dynamic) benchmarking platforms to provide relevant performance metrics, based on different use-cases, that would evolve together with the AMR field; second, agreed and defined datasets to allow the pipelines’ implementation, validation, and quality-control over time. Following previous discussions on the main challenges linked to this approach, we provide concrete recommendations and future steps, related to different aspects of the design of benchmarks, such as the selection and the characteristics of the datasets (quality, choice of pathogens and resistances, etc.), the evaluation criteria of the pipelines, and the way these resources should be deployed in the community.

## 1. Introduction

The technological advances in Whole Genome Sequencing (WGS) and the increasing integration of Next Generation Sequencing (NGS) platforms in the arsenal of testing laboratories is having a profound impact on health sciences. Affordable human genome sequencing is bringing about an era of improved diagnostics and personalised healthcare. For microorganisms, the reliable characterisation of their genetic material allows improved insights in their identity and physiology. Novel strategies for the implementation and analysis of NGS data are being developed and improved, and they can be used, for instance, to reconstruct the timeline and relationships between the cases of an infectious disease outbreak, which is something difficult to achieve with classical microbiological techniques.

Once sequenced, the genome of a microorganism can also be used to (re-)identify the species and infer important phenotypic properties, such as virulence, resistance to antibiotics, typing and other adaptive traits. An important aspect in this are considerations related to quality and consistency (see 1), in particular if the result of the method is to be used in a regulatory context (for example, in a monitoring framework) or, more importantly, in a clinical setting linked to decisions on medical treatments ^2– 4^, veterinary, agricultural or environmental interventions and food safety ^5, 6^ which may be linked under One Health initiatives.

Methods for predicting antimicrobial resistance (AMR) genetic determinants from NGS data rely on complex bioinformatics algorithms and procedures to transform the large output produced by the sequencing technologies into relevant information. Traditionally, regulatory implementation of analytical methods focuses on harmonisation of the protocol and the subsequent steps of analysis, i.e. ensuring the implementation of specific methods previously validated according to a set of criteria. For methods with important bioinformatics components, this is often not optimal, due to both the large variability in the developed strategies, variations in the particular computational resources available and the speed at which technologies and analytical approaches evolve. For the prediction of AMR determinants, very different strategies have been proposed, processing the sequencing data either as a set of reads or as pre-processed assemblies ^7, 8^, even using neural networks ^9^; sometimes, the system itself is proprietary and operates as a “black box” from the point of view of the user. In such cases like this, it has been proposed to approach the quality assurance challenge through performance-based evaluations, i.e. ensuring that the implemented methods, although different, perform at a similar (acceptable) level in this context ^10^. The same performance-based evaluation can then be applied whenever a component of the pipeline, or its environment, is replaced or updated.

An important component for a performance-based evaluation scheme is the availability of resources (in particular, datasets) that enable these evaluations ^11– 13^. In 2017, the Joint Research Centre (JRC) initiated a reflection on the subject by inviting experts in the field of AMR detection with NGS from the four compartments of a “One Health” perspective, i.e. clinics, food, animals and the environment ^14, 15^. These discussions led to a compilation of the challenges involved in the development of a benchmark strategy for bioinformatics pipelines, both for NGS-based approaches in general and in this specific field of application ^16^. These challenges were grouped into often overlapping categories, including the nature of the samples in the dataset (e.g. their origin, quality and associated metadata), their composition (e.g. the determinants and species to include), their use (e.g. expected results and performance thresholds) and their sustainability (e.g. their development, release and update).

On the 27 ^th^ and 28 ^th^ of May 2019, the JRC held a follow-up meeting, including most of the authors of the original article and additional experts that expressed interest, to discuss and propose solutions to the identified challenges for AMR detection using next generation sequencing. The present article represents a summary of these discussions and the conclusions reached. We propose this document as a baseline for a roadmap and guidelines to harmonise and standardise for the generation of the benchmark resources in the field of AMR.

## 2. Framing the aims and purposes of the benchmarking resources

An important observation that arose from the two-day discussions is that the concept of benchmarking, even when focusing on a single component of the method (i.e. the bioinformatics pipeline), may refer to different activities that can vary in their scope and objectives (see also 17, 18). Clarifying these scopes is crucial when proposing recommendations, as these (and the final datasets) will be influenced by the scope of the evaluation.

In the conclusions of the previous article, the use of the benchmark resources was reported as follows: *“(1) Ensuring confidence in the implementation of the bioinformatics component of the procedure, a step currently identified as limiting in the field. (2) Allowing evaluation and comparison of new/existing bioinformatics strategies, resources and tools. (3) Contributing to the validation of specific pipelines and the proficiency testing of testing laboratories and (4) “Future-proofing” bioinformatics pipelines to updates and replacement of tools and resources used in their different steps*.” ^19^.

These four summarising points made above, in practice, cover two different questions: 1, 3 and 4 (implementation, validation, proficiency testing and future proofing) ask whether the bioinformatics pipeline performs as expected, while 2 (evaluation/comparison) focuses on identifying gold standard pipelines and resources for implementation. The first scope of a benchmark resource would thus address the question: “Which pipeline performs best and at least by the agreed minimum standards?” A second scope addresses the question: “What is the quality of the information produced by the implemented bioinformatics pipeline?”

The latter question requires further refinement, based on the “what” the pipeline is “required” to achieve. Although there may be different contexts to the use of the methods (e.g. guide clinical intervention, contribute data to a monitoring framework, outbreak management, monitor the spread of AMR genes in or between different settings/environments, etc.), for a benchmark resource, these can be split in three main use cases:

- to predict the resistance of a pathogen of interest (either cultured or directly from samples of complex microbial communities)
- to identify the complete repertoire of the AMR determinants in a complex sample (i.e. the resistome)
- to identify the link of AMR genes to an specific taxon in a complex sample (i.e. taxon-binning approaches like described by Sangwan *et al.* ^20^).

Finally, another important scope for a benchmark resource was identified, having, once again, an impact on the decisions regarding the benchmark dataset: “How **robust** is the bioinformatics pipeline?” Studies addressing this question focus on identifying how the pipelines can tolerate variation in characteristics of the input data, most often related to the quality of the sample or sequencing steps: robustness against contamination or low number/poor quality reads, for example. Robustness, in certain contexts, could also be seen as the effect (or lack of) of swapping a tool (or the reference database) at a certain step in the pipeline for a different one that is functionally equivalent (see, for example, ^21^).

**Figure 1.**
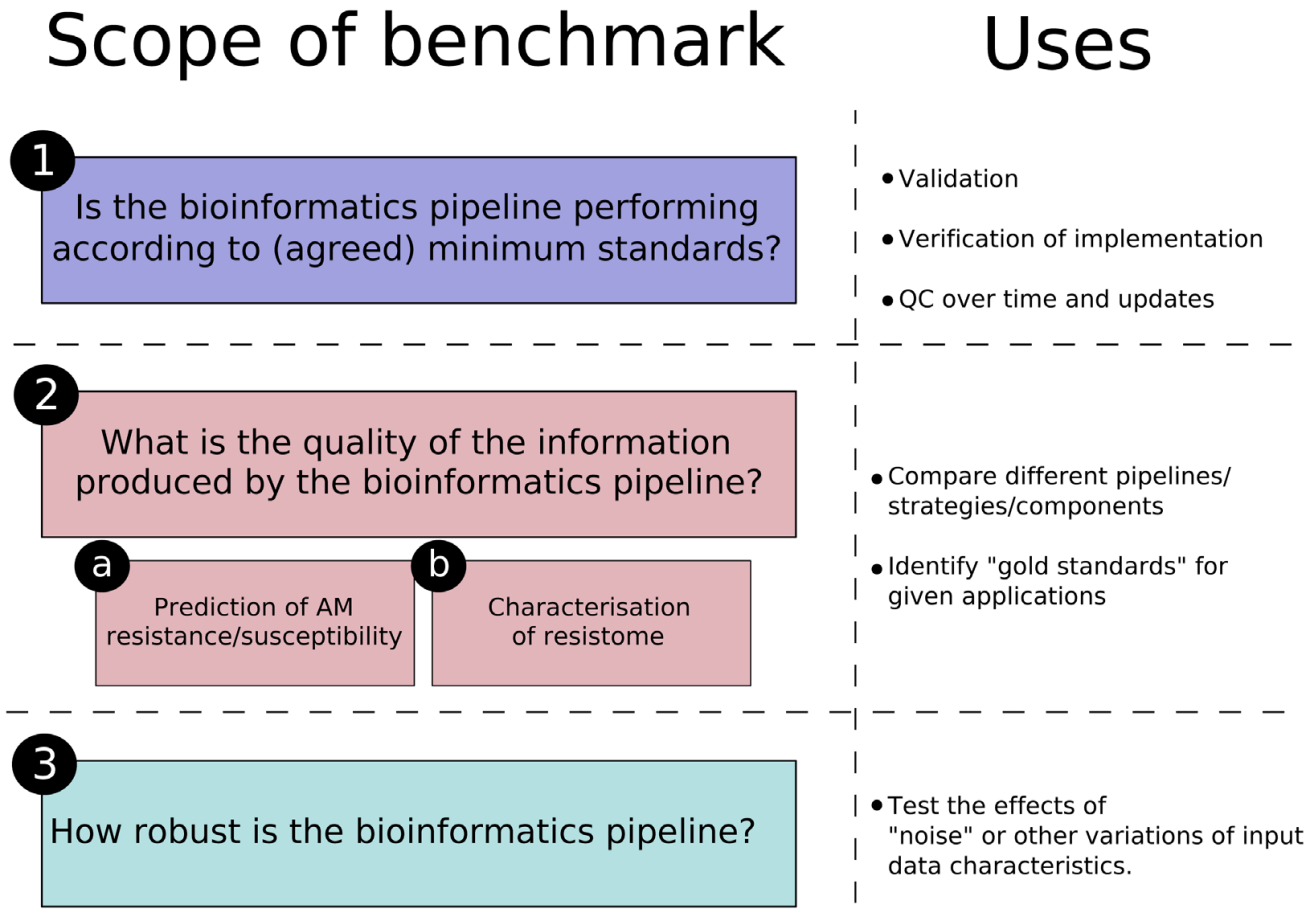
Summary of the different “scopes” for the benchmark resources for AMR detection using next generation sequencing discussed in the current document, with an indication of the uses for each.

In summary, it is important to be specific about the purpose and scope of the benchmark resource in the decisions taken when generating the datasets. We propose that the scope of a benchmark has three major parts, summarised in Figure 1.

### General considerations

When discussing the different challenges described in 16, rarely can an absolute “best” answer be identified for a given question; recommendations thus need to be made, taking into account the specific purpose of the benchmark resource and the fact that they may evolve with the state-of-the-art in the field.

Still, some general observations and conclusions were proposed, summarised in this section.

### 2.1. NGS platforms

A quick analysis of the different NGS platforms currently available and in development makes it obvious that the set of reads that they produce have very different characteristics. In addition, each platform has its strengths and weaknesses. Both the error rate (about 0.1% for Illumina (RRID:SCR_010233), 1–10% for newer technologies like from Pacific Biosciences and Oxford Nanopore Technologies (RRID:SCR_003756)) and the types of errors (miscalls, insertions or deletions, or problems of particular motifs such as homopolymer sequences) vary according to the platform used. The average length of the reads can vary from hundreds (Illumina, Ion Torrent) to thousands (PacBio, Nanopore) of base pairs ^22– 24^.

Bioinformatics pipelines are thus usually designed to handle the output of a specific platform, often in a certain configuration. Although exceptions exist (e.g. 25, 26), in the context of a benchmark resource (and independently of the question asked), we thus believe that different datasets are needed for each of the different NGS platforms, each containing reads that have a profile that matches closely with the normal output of the respective technologies. It is important, in this case, to ensure that the datasets produced for the different platforms do not introduce any bias among the different platforms (when bioinformatics pipelines analysing the output of different platforms are compared). Although the absence of bias may be hard to demonstrate *a posteriori*, efforts should be made to ensure that the datasets derive from strategies that are as similar as possible, for example by containing reads generated from the same input samples.

**Figure 2.**
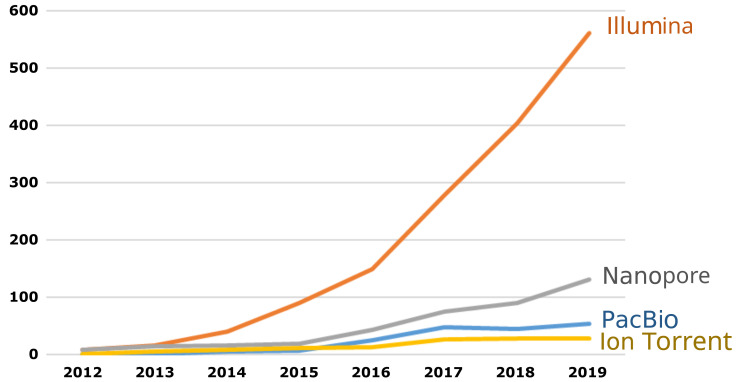
Number of articles published each year in the scientific literature mentioning the selected platform. Source: Scopus, using the search: ALL ( "X" AND "antimicrobial resistance" ).

The platforms for which benchmark datasets are produced should be selected based on pragmatic considerations. Ideally, equivalent resources should be available for all technologies; in practice, a prioritisation exercise should be made based on the capacity building efforts in testing laboratories. Recent surveys have shown a clear preference for the Illumina platform in this context ^27, 28^. The same trend can be observed when counting the number of published articles in a scientific literature database ( Figure 2).

The so-called “Third Generation Sequencing” technologies that sequence single DNA molecules and, importantly, produce long reads, have been shown to provide substantial benefits in the context of AMR detection. First, many resistance genes are located on plasmids, which are challenging to assemble using short-read sequencing technologies, because the short read lengths do not allow spanning of repetitive regions ^29^. The presence of an AMR determinant on a plasmid is also important for its transfer and eventual spread, and thus their correct assembly using long-read technologies represent a substantial advantage ^30– 34^. In addition, the proper and timely treatment of a pathogen infection is critical for successful prevention and control of diseases in clinical settings as well as in the community. In line with this, the Nanopore sequencing technology has shown the promise of providing accurate antibiotic resistance gene identification within six hours of sample acquisition ^35– 37^ We thus propose to include DNA Nanopore sequencing as an additional priority platform to develop benchmark resources.

The choice of formats for the different components of the datasets is also important. Each instrument produces a raw data output in a specific format (for example, the Illumina platforms generate raw data files in binary base call (BCL) format, while the Nanopore platforms produce FAST5 (HDF5) files). However, the entry point of most bioinformatics pipelines in this context is the description of the sequence of the produced reads, with an indication of the quality (or confidence) score for each of the base positions. The FASTQ format is a standard format in this context ^38^, which should be used in the benchmark resources; many tools exist to convert the raw data output files into this format in case of different platform outputs (see, for example, ^39, 40^) although, it should be noted, different tools may produce different results and this step should be carefully planned.

Other standard formats exist to describe intermediate states of processing, for example for the description of assembled contigs or variant calling ^41^. However, using these formats would make an *a priori* assumption about the strategy of the bioinformatics pipeline that may not be universal; indeed, not all reported solutions involve assembling reads, or mapping them to reference genomes or databases (see, for example, ^42, 43^).

### 2.2. Datasets origin

Three main sources of data for creating a benchmark dataset were identified. The first is to simulate the output (reads) *in silico* using an input sequence of a resistant pathogen and a specialised software. The second is to use the archived output of previously performed experiments that are available in different repositories. The third is to perform NGS experiments on biological samples.

Although the disadvantage of simulating *in silico* data is obvious (it is not ‘real’), there are some substantial advantages: it is a lot cheaper than performing sequencing runs, a lot faster, and can be applied to any genome previously sequenced. Thus, many more potential scenarios can be tested, for which the ground truth is well-established (i.e., the annotation of the genome reference that is used: different species, different classes of AMR, different localization of AMR), which usually cannot be done by actually sequencing them. Finally, it is also potentially ‘safer’ to do this for pathogenic bacteria for which high biosafety levels would be required to sequence in a laboratory. However, a major drawback is that simulating variation the way nature evolves is very challenging – genetic variation happens in places in the genome where it is hardest to find.

Many methods and programs have been developed to simulate genetic data. Their use in this context is, in itself, an exercise of Open Science and mechanisms should be used to guarantee quality and reproducibility (see 44) In 2013, Peng *et al.* ^45^ developed the catalogue “Genetic Simulation Resources” (GSR, available at https://popmodels.cancercontrol.cancer.gov/gsr/) to help researchers compare and choose the appropriate simulation tools for their studies. However, after reviewing the software listed in the GSR catalogue, the authors realised that the quality and usefulness of published simulation tools varied greatly due to inaccessible source code, lack of or incomplete documentation, difficulties in installation and execution, lack of support from authors and lack of program maintenance ^45^. For these reasons, a defined checklist of features that may benefit end users was defined ^46^; the “GSR Certification Program” was developed and recently implemented into the GSR in order to assess simulation tools based on these criteria ^47^. Established criteria are grouped to attribute four “certificates” ( https://popmodels.cancercontrol.cancer.gov/gsr/certification/):

- Accessibility: it ensures that the simulator is openly available to all interested users and is easy to install and use.
- Documentation: it ensures that the simulator is well documented so that users can quickly determine if the simulator provides needed features and can learn how to use it.
- Application: it ensures that the software simulator is peer-reviewed, is reasonably user-friendly to be useful to peer researchers, and has been used by researchers in the scientific community.
- Support: it ensures that the authors of the simulator are actively maintaining the simulator, addressing users’ questions, bug reports and feature requests.

As of December 2019, the GSR catalogue lists 148 simulators and many of them have been assessed for their compliance with the requirements in order to be certified. Obviously, not all of them are for simulation of NGS reads. In 2016 Escalona *et al.* ^48^ identified and compared 23 computational tools for the simulation of NGS data and established a decision tree for the informed selection of an appropriate NGS simulation tool for the specific question at hand.

By browsing the GSR catalogue, 20 out of 23 tools assessed by Escalona *et al.* (45) have been recorded, including only one with the four “GSR certificates” ( Table 1), i.e. the ART tool ^49^. Other tools not assessed by Escalona are also present in the GSR catalogue with certificates, like NEAT ^50^ and VISOR ^51^.

**Table 1.** Analysis of the GSR certifications of the computational tools for the simulation of next-generation sequencing described in 48. See text for details.

Tool	In GSR?	GSR certificate?			
		Accessibility	Documentation	Application	Support
*454sim*	Yes	not yet evaluated			
*ART*	Yes	Yes	Yes	Yes	Yes
*ArtificialFastqGenerator*	Yes	not yet evaluated			
*BEAR*	No	-			
*CuReSim*	Yes	No	Yes	No	No
*DWGSIM*	Yes	Yes	Yes	No	Yes
*EAGLE*	Yes	not yet evaluated			
*FASTQSim*	Yes	not yet evaluated			
*Flowsim*	No	-			
*GemSIM*	Yes	Yes	Yes	No	No
*Grinder*	Yes	not yet evaluated			
*Mason*	Yes	Yes	Yes	No	Yes
*MetaSim*	Yes	No	Yes	Yes	No
*NeSSM*	No	-			
*pbsim*	Yes	not yet evaluated			
*pIRS*	Yes	Yes	Yes	No	Yes
*ReadSim*	Yes	not yet evaluated			
*simhtsd*	Yes	not yet evaluated			
*simNGS*	Yes	not yet evaluated			
*SimSeq*	Yes	not yet evaluated			
*SInC*	Yes	Yes	No	No	Yes
*wgsim*	Yes	not yet evaluated			
*XS*	Yes	not yet evaluated			

For choice of the simulation methods and programs for NGS reads, the decision tree proposed by Escalona *et al.* is robust. However, it should be complemented by “certification” steps and, in this respect, we encourage the use of the “certification” criteria established by the GSR Certification Program, to tackle the challenge of following agreed principles for rigorous, reproducible, transparent, and systematic benchmarking of omics tools, in line with those proposed by Mangul *et al.* ^13^.

Using pre-existing experiments, from private or public repositories, ensures that the components of the dataset are representative of a real-life experiment, including the complete panel of real-life variabilities that are difficult to simulate. The main issues then are: a) there is a need to demonstrate that the experiment met the necessary quality criteria (see section 3.3); b) the “correct” value (i.e. the ‘ground truth’) for the experiment needs to be determined. This can be already described in the metadata associated with the record and/or determined (verified) *a posteriori –* although this requires strict annotation of the experiment; c) it will not be possible (besides rare exceptions) to build datasets for the different platforms using the same initial samples.

Generating experiments specifically for the sake of a benchmark dataset has almost the same advantages and disadvantages as using pre-existing data. Additional advantages include a better capacity to determine the “ground truth” of each sample by ensuring access to the original pathogen, as well as the possibility to generate datasets for the different platforms while using the same samples, if the same pathogen/purified DNA is processed through the different protocols and instruments. This also allows to better control of the quality aspects of the procedure performed, e.g. through the use of accredited laboratories who have therefore demonstrated by audits that they possess the necessary knowhow and expertise to create high-quality data. However, an additional disadvantage is that this process requires a substantial investment of time and resources (although this investment may be deemed worthwhile given the importance of the topic, and could benefit from the involvement of the instrument vendors).

Because each approach has advantages and disadvantages, the choice must be carefully considered, according to the purpose of the dataset, which will be discussed in section 4.

### 2.3. Quality metrics

The quality of the original sample and the wet laboratory procedures (e.g. DNA extraction, library preparation and sequencing) have a strong impact on the quality of the reads fed into the bioinformatics pipelines. Contamination, low amounts of reads passing the machine QC, higher error rates than normal, etc. can influence the output of bioinformatics pipelines. Usually, the pipelines are designed to be resilient to some extent to these variations.

Although understanding this resilience is important, we propose, as shown in Figure 1, to separate these considerations from resources meant for quality control and performance evaluation (questions 1, 2a and 2b) for two reasons: first, many of these factors are variable, heterogeneous, technology-specific, and can be implemented at different stages of the bioinformatics pipeline; attempting to incorporate them all in the same resource would be impractical and too costly. Second, pipelines implemented for regulatory or clinical decision-making will be incorporated into a larger quality assurance framework that will ensure the quality of the input until that step ^2^. Although examples exist of end-to-end NGS workflow validation (like in the case of WGS) where bioinformatics is one of the components ^52^, our approach emphasises on an approach where each step is validated separately (see 53).

It is then crucial to closely follow the proposed quality control schemes, either published or in development, in particular for the upstream steps (DNA isolation, library extraction, sequencing, etc.), for example ISO/TC 34/SC 9/WG 25. From these, both the metrics and the thresholds that can be applied at the level of the reads should be identified (some of which may vary according to the sequencing methodology), such as percent of bases with quality scores over Q30, percent alignment and error rates of the positive control (if present), the number of reads after trimming, etc. Tools exist that can provide a panel of quality metrics from FASTQ files, such as FASTQC (RRID:SCR_014583) ^54^. It is important to include the quality metrics as metadata in the dataset samples.

For the studies evaluating resilience (question 3), many different datasets as possible are needed for the “low quality dimensions” to be tested. For this reason, the establishment of standard datasets for this type of benchmarking is a complex exercise and answering question 3 of Figure 1 should be attempted on a case-by-case basis, thus it is better suited to individual studies. One way to harmonise the approach would be to use the datasets produced for questions 1 and 2 as a starting point, as there are tools that can add some extent of “noise” to existing good quality datasets ^49^.

### 2.4. Choice of bacteria/resistance to include

In the context of challenging/evaluating a bioinformatics pipeline for the detection of AMR genetic determinants, a very pragmatic approach could be the generation of random DNA sequences, to which particular sequences of interest are added (i.e. fragments of AMR genes). However, there is sufficient evidence that the genomic background of the bacteria (i.e. the “non-AMR related” sequences) can have a profound influence on the performance of the pipelines. For example, pipelines that include a contig assembly step will be affected by the frequency and level of repetitive sequences in the background genome, as well as its GC content ^55, 56^. Some species also have genes that are similar at the sequence level to known AMR determinants that efficient pipelines must be able to distinguish.

In conclusion, the bacterial species included in the benchmark datasets, and the AMR genes they contain, need thus to be carefully selected, with the appropriate justifications. These are specific to the purpose of the dataset ( Figure 1) and will be discussed in section 4.1–section 4.3 below.

### 2.5. Genomic or phenotypic endpoint

A pipeline processing sequencing information for AMR can produce two closely linked but conceptually different outputs: a) they can detect the genetic determinants of AMR, and in addition b) some can predict the AMR/susceptibility of the bacteria in the original sample.

In a clinical context, the phenotypic endpoint is the most relevant, as it provides the information most useful for the end users. Studies that evaluated AMR genotype to phenotype relationships have indicated that despite generally high correspondence, this can vary greatly between pathogens / case studies, and even for different antimicrobial agents within the same species ^57, 58^. There are different reasons for discrepancies between phenotype and genotype, including the extent of the expression of the resistance determinants in order for the resistance to be conferred, and also relatively complex molecular pathways that can influence the eventual phenotype. In some cases, genes can also confer reduced susceptibility (i.e. increasing the concentration of an antimicrobial necessary for treatment) rather than resistance *per se*. A genotypic endpoint may also be problematic due to the definition of “antibiotic resistance” in different settings ^59^, which can complicate the interpretation of results.

In practice, however, focusing on a genomic endpoint has many advantages:

- The end-point (determinant; gene or SNP) is better defined: presence or absence.
- The gene copy number can be calculated, this is important even if obtaining gene copy numbers with short read data remains pretty difficult.
- It provides high resolution information, that is useful when many genetic determinants confer resistance to the same antimicrobials.
- It offers additional information to contribute to an evaluation of the history of the spread of AMR ^60^.
- It does not rely on breakpoints such as Minimum Inhibitory Concentrations (MICs), which may vary between human and animal bacterial isolates, or may not be available for some animals (or pathogens), or because it may be updated based on phenotypic scientific observations ^61, 62^.
- Even in the cases of AMR determinants not being expressed (thus not leading to a resistance phenotype), this may be important to characterise/record for epidemiological purposes.

### 2.6. Benchmark datasets metadata

Besides the set of reads themselves, additional information needs to be associated with each sample in the dataset.

Obviously, each sample needs to include a “true” value, i.e. the ‘ground truth’ to be used for comparison when evaluating the performance of the pipeline. For a genotypic endpoint, this would take the form of the name (with a reference to a database) of the AMR determinants present. If real-life samples are used, the phenotypic information should be included, on top of the genotypic endpoint.

Public resources and repositories are available to host both the data and the metadata, and should be used as appropriate for the sake of transparency, obsolescence and traceability of the datasets of the benchmark resource. In practice, this means:

- The NGS reads data should be hosted in one of the International Nucleotide Sequence Database Collaboration (INSDC, RRID:SCR_011967) sequence reads archives ^63^, compiling the metadata information as appropriate.
- For simulated reads, this information should include the simulation tool used (source, version, parameters).
- For simulated reads, the “input” sequence(s) should be a closed genome, and any additional genes, that should be available in INSDC sequence archives ^64^, and the record ID(s) included in the reads metadata information. Optimally, the closed genomes should be linked to a “real” sample in the INSDC BioSample database.
- For real experiments, the originally sequenced sample should be present/submitted in the INSDC BioSample database ^65^, with all the appropriate metadata information (including identified resistances and the MIC(s) determined according to the standard culture-based evaluation methods).

## 3. Design of the scope-specific benchmark resources

### 3.1. Is the bioinformatics pipeline performing according to (agreed) minimum standards?

The scope of this benchmark resource is to address the questions of validation, implementation and quality control over time (i.e. following any change in the pipeline or the environment on which it is executed). The dataset required for this should be compiled based on an agreed “minimum” standard, i.e. thresholds for the acceptance values of certain performance metrics for the bioinformatics pipeline in the context of the detection of AMR determinants, no matter the exact final use of the information produced.

This evaluation of performance should be based on challenging the pipeline with input representing a carefully selected set of resistance determinants and bacterial hosts. These sets of NGS reads should be fully characterised regarding their genetic content and serve as ( *in silico*) reference materials for the validation and quality control of the bioinformatics component of the methods (see, for other host models, 66, 67).

To maintain this necessary control on the genetic content of the reads, the dataset should be composed exclusively of simulated experiments. Nevertheless, this does not exclude at all the use of real data, which would be extremely relevant for cases like when the presence/absence of some AMR determinants has been established using first generation of consolidated and classical molecular-biology-based methods (e.g. PCR + Sanger sequencing). Synthetic reads can be generated in a large scale, in a harmonised manner, and most importantly allow full control on the content of the output. For the choice of resistances and bacterial species to be included, it is proposed to select them based on three sources, based on their current public health relevance and regulatory frameworks:

- The WHO’s list of antibiotic-resistant "priority pathogens" ^68^.
- The AMR reporting protocol for the European Antimicrobial Resistance Surveillance Network (EARS-Net) ^69^.
- The Commission Implementing Decision of 12 November 2013 on the monitoring and reporting of AMR in zoonotic and commensal bacteria ^70^.

Table 2 shows the combination of these three lists, in terms of both the bacterial species and the antibiotics mentioned.

**Table 2.** Summary of the bacterial species and antibiotics resistances mentioned in the three lists discussed in the text. a: WHO’s list of antibiotic-resistant "priority pathogens". b: EARS-Net reporting protocol for 2018. c: Commission Implementing Decision 2013/652/EU.

	*Acinetobacter baumannii*	*Campylobacter coli*	*Campylobacter jejuni*	*Enterococcus faecalis*	*Enterococcus faecium*	*Escherichia coli*	*Haemophilus influenzae*	*Helicobacter pylori*	*Klebsiella pneumoniae*	*Neisseria gonorrhoeae*	*Pseudomonas aeruginosa*	*Salmonella spp.*	*Shigella spp.*	*Staphylococcus aureus*	*Streptococcus pneumoniae*
*Amikacin*	b					b			b		b				
*Amoxicillin*				b	b	b			b						
*Ampicillin*				b,c	b,c	b,c	a					c			
*Azithromycin*						c						c			b
*Cefepime*						b			b		b				
*Cefotaxime*						b,c			b			c			b
*Cefoxitin*														b	
*Ceftazidime*						b,c			b		b	c			
*Ceftriaxone*						b			b						b
*Cephalosporin*										a					
*Chloramphenicol*				c	c	c						c			
*Ciprofloxacin*	b	a,c	a,c	c	c	b,c			b	a	b	a,c	a	b	
*Clarithromycin*								a							b
*Cloxacillin*														b	
*Colistin*	b					b,c			b		b	c			
*Daptomycin*				c	c									c	
*Dicloxacillin*														b	
*Ertapenem*	a					a,b			a,b		a	a	a		
*Erythromycin*		c	c	c	c										b
*Flucloxacillin*														b	
*Gentamicin*	b	c	c	b,c	b,c	b,c			b		b	c			
*Imipenem*	a,b					a,b			a,b		a,b	a	a		
*Levofloxacin*	b	a	a			b			b	a	b	a	a	b	b
*Linezolid*				b,c	b,c									b	
*Meropenem*	a,b					a,b,c			a,b		a,b	a,c	a		
*Methicillin*														a,b	
*Moxifloxacin*						b			b						b
*Nalidixic acid*		c	c			c						c			
*Netilmicin*	b					b			b		b				
*Norfloxacin*						b			b					b	b
*Ofloxacin*		a	a			b			b	a		a	a	b	
*Oxacillin*														b	b
*Penicillin*															a,b
*Piperacillin*						b			b		b				
*Polymyxin B*	b					b			b		b				
*Quinupristin/ Dalfopristin*				c	c										
*Rifampin *														b	
*Streptomycin*		c	c												
*Sulfamethoxazole*						c						c			
*Teicoplanin*				b,c	b,c										
*Tetracycline*		c	c	c	c	c						c			
*Tigecycline*				c	c	b,c			b			c			
*Tobramycin*	b					b			b		b				
*Trimethoprim*						c						c			
*Vancomycin*				b,c	a,b,c									a,b	

In practice, the simulated reads should be derived from:

1. High-quality and complete reference genome sequences for the pathogens in Table 2. See, for example, the FDA-ARGOS database ^11^ and the NCBI RefSeq Genomes database (RRID:SCR_003496).
2. Known genetic determinants for the resistance against the antibiotics in Table 2, using available resources ^7, 8, 71^. If more than one determinant is associated with a resistance phenotype, one possibility is to collect them all; expert knowledge and empirical evidence on the relative contribution of different genes to the phenotypes, from published large-scale studies (e.g. 72) can also be used to objectively reduce the list of determinants to include for a given antibiotic.
3. Combinations of (1) and (2) present in at least one of the chosen lists (see cells in Table 2), the sequences are combined and used as the input to simulate the reads using the appropriate tools (see section 3.2).

It is important to highlight that the combination of these three lists still leaves important regulatory gaps, and should be complemented by the World Organisation for Animal Health (OIE, RRID:SCR_012759) list of antimicrobial agents of veterinary importance ^73^ or others ^74^. However, the lists do not mention specific species associated to each antibiotic, and these should be selected by the appropriate experts for the context of this benchmark resource.

The endpoint considered for this benchmark is thus genotypic (see section 3.5), and the main metric measured is the pipeline’s accuracy in the identification of the correct genetic determinants or alleles.

Because of the selection of this subset of bacteria/resistances and their immediate clinical and regulatory importance, an important performance metric to be evaluated with this dataset is accuracy. The reference genomes for each pathogen used to simulate the reads should be carefully chosen and characterised to ensure all present AMR determinants (besides the one that will be added to the sequence prior to simulating the reads) are carefully recorded to avoid unfair assignment of “false positives” to the pipelines that will (correctly) identify them.

There are other important performance metrics to consider in the context of validating a bioinformatics pipeline, such as repeatability, reproducibility, sensitivity, specificity, and precision, whose definitions need to be carefully considered in this context ^53^ (for example, “reproducibility”, could be evaluated as the result of running the same bioinformatics pipeline, with the same datasets, implemented in different systems).

For all the performance metrics, the minimum acceptable values should be subsequently determined once the outputs of real benchmarking exercises considering all the aspects described in this article are available.

When generated, the benchmark should be deployed on a dedicated (and sustainably maintained) platform that includes all the links to the data (see section 3.6) and a description of all the steps/decisions that were taken to generate it. It is also important to implement, from the start, a clear version control system for the benchmark resource, in order to properly document the changes over time, and the exact versions used at the different times that the resource is used. In addition to the unique accession numbers of the individual samples in their respective repositories, the dataset as a whole should have a unique identifier (e.g. a DOI) that changes when any modification is made. The versioning should also allow access to and use of any previous versions of the resource, even after being updated.

This minimal dataset contains, by definition, a limited number of species and may lack pathogens of clinical importance (for example, *Mycobacterium tuberculosis*, for which WGS-based approaches have shown particular advantages, see 75, 76). A full validation exercise for a specific pipeline, applied to a specific context, will need additional samples that complement the resource described in this section with the appropriate species/resistances. These datasets may, for example, be taken from the resources described in the following sections, that focus on evaluating the actual performance of methods in broader contexts by gathering the many datasets necessary to do so.

### 3.2. What is the quality of the information produced by the bioinformatics pipeline (prediction of resistance)?

The scope of this benchmark resource is to identify gold standards for bioinformatics pipelines, in this case linked to the specific use of predicting resistance/susceptibility of a pathogen.

There is a step between identifying the determinants of AMR and predicting resistance, which is not always straightforward as factors such as expression of the AMR gene may affect the prediction ^57, 72^. For this reason, and because it is conceptually closer to the information that is acted upon, the endpoint for this benchmark should be phenotypic. In addition, the dataset should be composed of real NGS experiments, since artefacts and variations are more complex in real sequencing reads than in simulated reads, a factor crucial to consider for this scope that focuses on accuracy.

To minimise the need of extensive resources to produce these “real” datasets, we propose to focus on re-using experiments previously performed under standardised conditions. A great source of data are the published ring trials; these have the additional advantage of providing an accurate characterisation of the sequenced samples, since the same original samples are sequenced many times by different laboratories. If needed, the data generated by single-site studies can also be evaluated, although in this case the issue of the correct characterisation of the samples (their “true” resistance patterns) should be addressed. One possibility is to use studies performed in a hospital setting, linked to clinical outcome (for example, ^77^), or where sufficient information is available to evaluate the way the susceptibility testing was performed.

In practice, this would mean:

1. Performing an extensive review of the published literature to identify studies, ring trials, and proficiency testing that meet the criteria (focused on the detection of AMR using NGS, starting from a “real” sample). Table 3 provides a non-exhaustive list of recent references to be used as a starting point.
2. Assessing whether the raw sequencing output for the projects meet the FAIR principles (Findability, Accessibility, Interoperability, and Reusability) ^78^, and are retrievable from publicly available repositories – even if they are access controlled. If not fully open, the corresponding authors should be contacted and asked whether the data could be obtained and deposited in long-term archives (e.g. Zenodo (RRID:SCR_004129), EuDat and/or the European Nucleotide Archive (ENA, RRID:SCR_006515) depending on the deposited data).

**Table 3.** Sample studies to be analysed for the availability of FAIR raw reads data, to include in the benchmark resource.

Pathogens	Study	Year	Study type	Ref
*Clostridium (Clostridioides) difficile*	Berger *et al.*	2019	ring trial	79
*Neisseria meningitidis*	Bogaerts *et al.*	2019	validation study	53
*Salmonella enterica*	Mensah *et al.*	2019	single study	80
*Enterobacteriales*	Ruppé *et al.*	2019	single study	58
*Escherichia coli*	Stubberfield *et al.*	2019	single study	81
*Staphylococcus aureus*	Deplano *et al.*	2018	ring trial	82
*Brucella melitensis*	Johansen *et al.*	2018	ring trial	83
*Salmonella enterica*	Neuert *et al.*	2018	single study	84
*Salmonella, Campylobacter*	Pedersen *et al.*	2018	proficiency testing	85
*Escherichia coli*	Pietsch *et al.*	2018	single study	86
*Enterococcus faecium, Enterococcus faecalis*	Tyson *et al.*	2018	single study	87
*Actinobacillus pleuropneumoniae*	Bossé *et al.*	2017	single study	88
*Klebsiella pneumoniae*	Brhelova *et al.*	2017	single study	89
*Salmonella enterica*	Carroll *et al.*	2017	single study	90
*Escherichia coli*	Day and al.	2016	single study	91
*Salmonella* spp. *, Escherichia coli, Staphylococcus aureus*	Hendriksen *et al.*	2016	proficiency testing	92
*Salmonella*	McDermott *et al.*	2016	single study	93
*Staphylococcus aureus, Enterococcus faecium, Escherichia coli,* *Pseudomonas aeruginosa*	Mellmann *et al.*	2016	single study	77
*Staphylococcus aureus, Mycobacterium tuberculosis*	Bradley *et al.*	2015	single study	42
*Escherichia coli*	Tyson *et al.*	2015	single study	94
*Mycobacterium tuberculosis*	Walker *et al.*	2015	single study	75
*Campylobacter jejuni, Campylobacter coli*	Zhao *et al.*	2015	single study	95
*Pseudomonas aeruginosa*	Koos *et al.*	2014	single study	96
*Staphylococcus aureus*	Gordon *et al.*	2013	single study	97
*Escherichia coli, Klebsiella pneumoniae*	Stoesser *et al.*	2013	single study	98
*Staphylococcus aureus, Clostridium difficile*	Eyre *et al.*	2012	single study	99
*Salmonella* typhimurium *, Escherichia coli, Enterococcus faecalis,* *Enterococcus faecium*	Zankari *et al.*	2012	single study	100
*Salmonella*	Cooper *et al.*	2020	Single study	101

These datasets would then be used to test and compare the different bioinformatics pipelines in order to calculate the accuracy of their phenotypic predictions. Although not exhaustive, these datasets should cover the most relevant “real-life” cases, as they warranted their inclusion into a ring trial, with the associated resources committed to produce the data. The final size and composition (species, resistances) of the dataset would depend on what is provided by the available projects; *ad hoc* ring trials could be organised to cover eventual important gaps in species and/or resistance.

Although the chosen endpoint is mostly phenotypic, the purpose is to evaluate bioinformatics pipelines that process information at the sequence level, so it was agreed that there was little added value of inserting resistant samples (based on a characterised or inferred phenotype) for which the resistance mechanism is still unknown. In any case, it is improbable that these cases would have been included in ring trials projects.

Although the performance metrics described in Section 4.1 apply and are relevant in this case, the main performance metric for this benchmark is the accuracy. Because of the difficulty of predicting the link between the presence of AMR determinants and their impact on the pathogen susceptibility to the antimicrobial agents, the target accuracy is expected to be lower than for a genotypic endpoint. Both false positives and false negatives can be an issue when the information is used for clinical intervention, so a sufficient amount of “borderline” cases should be included, and both sensitivity and specificity evaluated. It is also possible to consider attaching different relative costs for false positives and false negatives when evaluating the accuracy metrics.

Once selected and combined, the data should be separated by NGS platform, and by species and antibiotic. Because this benchmarking aims at evaluating and comparing performance of methods, which are continuously developed and optimised, against a large and constantly expanding dataset, it is crucial to define an environment where the AMR community can establish a continuous benchmarking effort. Within this platform, pipelines would be compared simultaneously based on up-to-date datasets, under the same conditions, and over time. Constantly updating and adding to the reference datasets is important both to keep up with the evolution of the knowledge/reality in the field, and to avoid that pipelines are developed that are optimised to specific datasets only.

One option is OpenEBench (Open ELIXIR Benchmarking and Technical Monitoring platform), which is developed under the ELIXIR-EXCELERATE umbrella in order to provide such a platform ^18^. In this framework, in addition to compiling the data resources to be included (as described above), and whatever the platform chosen, there will be the need for efforts to:

- Establish guidelines for input and output formats (and, in the case of the phenotypic endpoint, an agreed ontology for the conclusions).
- Encouraging a “FAIR” implementation of the pipelines themselves, to increase the number of pipelines accessible for the benchmarking platform, and for interested end users to retrieve and implement in house.

Provisions should be included to allow the possibility to evaluate, in this context, pipelines that cannot be made “FAIR” based on intellectual property rights, institutional policies or available resources.

A final step will be to communicate these efforts within the scientific community and the potential end users, as well as to demonstrate the added value of this “live” benchmark resource to ensure that future studies (in particular, their pipelines and the datasets they generate) are efficiently integrated in the platform.

### 3.3. What is the quality of the information produced by the bioinformatics pipeline (mixed samples)?

Many gaps exist in the scientific understanding of antibiotic resistance development and transmission, making it difficult to properly advise policy makers on how to manage this risk. There is strong evidence that a multitude of resistance genes in the environment have not yet made it into pathogens ^102, 103^; understanding the relative importance of different transmission and exposure routes for bacteria is thus crucial ^59, 104– 106^.

Establishing a baseline for resistance determinants in the environment, and linking this to a surveillance scheme, requires a good understanding of the relative performance of methods that are and have been developed to characterise the resistome in a complex sample. There would be, also for this use case, a great value in the establishment of a community-driven “live” benchmarking using a platform such as OpenEBench, and many of the concepts that were discussed in section 4.2 apply here as well, with the following differences:

- As, by definition, the resistome refers to the genetic determinants (and not directly the associated phenotypes) ^107, 108^, the endpoint for this benchmark should be genotypic.
- Culture-dependent methods established for clinical samples cannot always be readily applied to environmental samples ^109^, so establishing “true” values for real samples, to compare the output of the evaluated pipelines, will be difficult, so the benchmark should be performed, at this stage, with simulated reads.

The resistome is usually derived from a sample containing a complex microbial community (see 110– 112 for recent examples). For this reason, the approaches ^113^ and tools ^114^ from the ongoing Critical Assessment of Metagenome Interpretation (CAMI) could be considered when organising the community around this challenge.

In practice, this means an effort to engage and coordinate the community of bioinformatics pipelines designed to predict the resistome of a sample in order to:

1. Design the scope of the challenge, including the relevant metrics for performance evaluation. For this, “accuracy”, the main metrics for the previous two benchmarks, may not be the most appropriate, and the focus should be placed, e.g., on “recall” and “precision”.
2. Describe the microbial communities (i.e. microbial abundance profiles and their typical AMR gene profiles) most relevant for the determination/monitoring of the resistome, in order to generate congruent datasets that accurately represent real-life samples. Of particular interest, for which validation will eventually be a prerequisite, are blood, serum, saliva etc., i.e. the types of samples clinical microbiology laboratories and national reference centres/laboratories typically process.
3. Identify both the microbial genomes and the resistance determinants (as single genetic determinants or plasmids) necessary to generate the profiles identified in (2). As stated in section 4.1, the genomes should be well analysed to ensure no lack of, or an adequate characterisation of, AMR determinants. This is crucial in order to establish a resistome “ground truth” for the generated datasets.
4. Combine these sequences, as appropriate, to generate the benchmark datasets, using appropriate tools (such as CAMISIM, developed as part of the CAMI challenge ^114^).

The community should decide whether (or at what stage) the use of real data can also be considered in the challenge. As for purified bacteria (see Table 3), many studies have been published as potential sources of raw data. These studies can also be used as a source of information to define the relevant profiles (point 2 above). Recent studies include resistome determination in samples from drinking water ( 115, 116), wastewater plants ( 117, 118), hospital wastewater ( 119, 120), human gut ( 110, 121), sewage ^122^, to name a few. In the benchmarking platform, the datasets (and the calculated pipeline performances) should be separated by the type of source they originate from or simulate. Another important point for this scope is the detection of minority populations and the correct normalisation of the samples to be analysed ^123^.

## Conclusions

The scientific community quickly adopted the new NGS technologies to develop methods that can efficiently detect, identify and characterise genetic determinants of AMR. In parallel with these research uses, NGS technologies can have immediate impacts on how AMR is diagnosed, detected and reported worldwide, complementing etiologic agent diagnosis, clinical decision making, risk assessment and established monitoring frameworks ^3, 124– 126^.

For this application and in general, there are great challenges in the implementation of NGS-based methods for public decision-making. Capacity building and its cost is of course a factor, but recent surveys show that capacity development is ongoing in many countries ^28^. A greater concern is the interpretation of the produced genomic data into meaningful information that can be acted upon or used for regulatory monitoring, in great part because of the important bioinformatics component of these methods.

The difficulties posed by this reliance on bioinformatics processes are many, and include:

- The specific expertise needed for their implementation and maintenance, which is still limited compared to the needs of routine testing environments.
- The lack of harmonisation in their design, as the same sequencer output can be processed to produce the same target information by pipelines that either follow the same general strategy, with different tools for the individual steps, or completely different strategies entirely (see 127).
- The constant, rapid evolution of the fields of informatics and bioinformatics, which makes uneasy (or even unwise) to “freeze” a harmonised, validated, implemented pipeline with the same components in the same environment over long periods of time.
- For AMR, as for other fields, the pipelines (and their performance metrics) are built based on *a priori* scientific knowledge, in this case the genetics of resistance, which is constantly progressing.

In this document, we propose a way through these difficulties with a transparent, performance-based evaluation approach to assess and demonstrate that pipelines are fit-for-purpose and to ensure quality control. The discussions, initiated in 2017 ^16^, have involved experts in different fields: human health, animal health, food and environmental monitoring, and general bioinformatics.

The approach is two-fold: first, an agreed-upon, limited dataset to contribute to performance-based control of the pipeline implementation and their integration in quality systems. We propose selection criteria for this common dataset based on bacterial species and resistances relevant to current public health priorities (see section 4.1).

Second, a community-driven effort to establish a “live” benchmarking platform where both the datasets and the bioinformatics workflows are available to the community according to the FAIR principles. After an initial investment of resources to establish the rules and integrate the existing resources, a proper engagement of the community will be needed to ensure that both the datasets and the workflows will constantly be updated, with live monitoring of the resulting comparative performance parameters. For this, two main use cases were identified, each necessitating its own platform: the analysis of isolates (with a focus on the prediction of resistance, see section 4.2), and the analysis of mixed samples (with a focus on the interpretation of the resistome, see section 4.3).

To ensure acceptance of this approach by regulators and policy-makers, the conclusions and the roadmap proposed in this document should be complemented (and, if necessary, revised) with the continuous involvement of all relevant actors in the field, including (but not limited to) the scientific community, the collaborative organisation and platforms active in the field (e.g. the European Committee on Antimicrobial Susceptibility Testing (EUCAST), the Joint Programming Initiative on Antimicrobial Resistance (JPIAMR), the Global Microbial Identifier (GMI), the European Society of Clinical Microbiology and Infectious Diseases and its Study Groups (ESCMID)), regulatory agencies (e.g. the European Food Safety Authority (EFSA, RRID:SCR_000963), the European Centre for Disease Prevention and Control (ECDC)), European Union reference laboratories and their networks (e.g. the EURL AR and the EURLs for the different pathogens) and the existing bioinformatics infrastructures (e.g. the European Bioinformatics Institute (EMBL/EBI), ELIXIR).

Such an approach would be a way to facilitate the integration of NGS-based methods in the field of AMR, and may be a case study on how to approach the overlapping challenges in other potential fields of applications, including some at high level in policy agendas (food fraud, genetically modified organism detection, biothreats monitoring for biodefense purposes, etc.).

## Disclaimer

The contents of this article are the views of the authors and do not necessarily represent an official position of the European Commission or the U.S. Food and Drug Administration.

## Data availability

No data is associated with this article.
