# Supplementary material for: A roadmap for the generation of benchmarking resources for antimicrobial resistance detection using next generation sequencing
Source: F1000Res. 2022 Mar 16;10:80. Originally published 2021 Feb 8. [Version 2] doi: 10.12688/f1000research.39214.2 (PMC9243550; doi:10.12688/f1000research.39214.2)
Supplement: Peer review history

# Peer review: Reviewer response for version 2

**DOI:** 10.5256/f1000research.122296.r135212

**Published:** 2022-06-29

**Authors:** Enrico Lavezzo, Emilio Ispano

**Affiliations:** 1 Department of Molecular Medicine, University of Padova, Padua, Italy

The manuscript “A roadmap for the generation of benchmarking resources for antimicrobial resistance detection using next generation sequencing” by Petrillo and coauthors addresses a crucial issue, which is the need for a standard benchmark dataset to assess bioinformatics pipeline performing AMR detection/prediction. The logic followed by the authors to produce guidelines to build such benchmark dataset(s) is sound and clear, and so are the discussion and conclusion. Although the paper is well-written and holds solid evidence, and surely meets the need of the scientific community to have a gold standard for this topic, there are some paragraphs that could be adjusted for clarity and they will be listed below. In addition, it is not clear why past attempts to create such databases are not mentioned, since it could provide a much more advanced starting point than building it from scratch. I am referring to McArthur, A.G *et al.* (2013) ^1^ (which introduces also an ontology to represent AMR, could it serve the purpose addressed in the last bullet of section 3.2?), Zankari, *et al.* (2012) ^2^.

**Abstract**

- "For this application and in general, considerable challenges remain in demonstrating sufficient trust to act upon the meaningful information produced from raw data…", meaningful but convoluted sentence, could benefit from rephrasing.

**Introduction**

- First paragraph: A reference could be added to support the stated implementation of strategies using NGS to track the timeline and relationships between cases of an outbreak.
- Third paragraph: "For methods with important bioinformatics components…", what do you mean by important? That the bioinformatic component is crucial for the method? Or that the component is by itself complex and structured?
- Third paragraph: "in such cases like this…" is a repetition. I suggest using just "In such cases".

**Section 2.3**

- Second paragraph: from "Although understanding…" to "…too costly" is a sentence longer than needed, I suggest a full stop instead of the colon.

**Section 2.4**

- First paragraph: "Some species also have genes that are similar at the sequence level to known AMR determinants that efficient pipelines must be able to distinguish.", this could benefit from some references pointing to such species.
- Second paragraph: The authors imply the need to cut several bacterial species from the database, which is nonsense in the scope of an ideally global benchmark database. I would rather include as much information as possible and add metadata to characterise every database entry to discern whether to use it or not for a certain evaluation.

**Section 2.6**

- First paragraph: "Besides the set of reads themselves, additional information needs to be associated with each sample in the dataset, not for the benchmarking per se but its use for next benchmarking exercises", this sentence is hard to understand, please rephrase.
- Third paragraph: "and should be used as appropriate for the sake of transparency", what do the authors mean?

**Section 3.1**

- Fourth paragraph: Check also Murray (2022) ^3^ as it provides comprehensive data and estimations regarding many AMR cases worldwide.

**Section 3.2**

- Third paragraph: "A great source of data are the published ring trials…". Explain what a ring trial is since it is also a key step for the understanding of Table 3.
- Last bullet: I am again pointing to McArthur, A.G *et al.* (2013) ^1^ for such ontology.

Is the topic of the opinion article discussed accurately in the context of the current literature?

Yes

Are arguments sufficiently supported by evidence from the published literature?

Partly

Are all factual statements correct and adequately supported by citations?

Partly

Are the conclusions drawn balanced and justified on the basis of the presented arguments?

Yes

Reviewer Expertise:

Microbiology, bioinformatics, genetics

We confirm that we have read this submission and believe that we have an appropriate level of expertise to confirm that it is of an acceptable scientific standard, however we have significant reservations, as outlined above.

## References

[1] : The comprehensive antibiotic resistance database. *Antimicrob Agents Chemother* .2013;57(7) : 10.1128/AAC.00419-13 3348-57 10.1128/AAC.00419-13 23650175PMC3697360

[2] : Identification of acquired antimicrobial resistance genes. *J Antimicrob Chemother* .2012;67(11) : 10.1093/jac/dks261 2640-4 10.1093/jac/dks261 22782487PMC3468078

[3] : Global burden of bacterial antimicrobial resistance in 2019: a systematic analysis. *The Lancet* .2022;399(10325) : 10.1016/S0140-6736(21)02724-0 629-655 10.1016/S0140-6736(21)02724-0 PMC884163735065702

# Peer review: Reviewer response for version 2

**DOI:** 10.5256/f1000research.122296.r127727

**Published:** 2022-05-13

**Authors:** Anna Abramova, Marcus Wenne

**Affiliations:** 1 Department of Infectious Diseases, University of Gothenburg, Gothenburg, Sweden

We thank the authors for taking into consideration our comments and suggestions.

Is the topic of the opinion article discussed accurately in the context of the current literature?

Yes

Are arguments sufficiently supported by evidence from the published literature?

Yes

Are all factual statements correct and adequately supported by citations?

Yes

Are the conclusions drawn balanced and justified on the basis of the presented arguments?

Yes

Reviewer Expertise:

AMR, NGS data analysis, bioinformatics

We confirm that we have read this submission and believe that we have an appropriate level of expertise to confirm that it is of an acceptable scientific standard.

# Response

**Published:** 2022-05-13

**Authors:** Mauro Petrillo

**Affiliations:** European Commission, Joint Research Centre (JRC), Ispra, Italy

Dear Dr Abramova and Dr Wenne,

 We would like to thank you for your valuable comments and suggestions which definitively improved the quality of our manuscript.

 Best regards,

 Mauro Petrillo, on behalf of the authors.

# Peer review: Reviewer response for version 1

**DOI:** 10.5256/f1000research.42277.r91069

**Published:** 2021-09-14

**Authors:** Anna Abramova, Marcus Wenne

**Affiliations:** 1 Department of Infectious Diseases, University of Gothenburg, Gothenburg, Sweden

Petrillo and colleagues discuss approaches and associated challenges for establishing a performance-based benchmarking resource for antimicrobial resistance detection. The authors first describe general considerations and then provide scope-specific use cases. The paper represents a summary of the discussions held by experts in AMR and NGS-related fields during the JRC meeting. It is a relevant and important paper, and the initiative will be widely appreciated by the scientific community working with antimicrobial resistance. Overall, the paper is well-written, however, it would benefit from some additional clarifications and adjustments, which are explained in more detail in the comments below.

**Introduction**

- Since the main focus of the paper is benchmarking, it would be beneficial to provide a short background on the previous benchmarking resources/initiatives in the introduction (e.g. Mangul *et al*., 2019 ^1^, Sczyrba *et al*., 2017 ^2^, etc).

**Section 2 **

- Paragraph 2: “In the conclusions of the previous article...”, it is confusing which article the authors refer to since reference 19 (Bellman *et al*., 2015) provided at the end of the sentence does not contain the cited text.

**General considerations**

- Several important questions such as which tools to include in the benchmarking, should they be run with optimised or default parameters (e.g. default or customised database), and what performance metrics are to be used for evaluation - could be added to the "General discussion" to clarify and benefit in the understanding of the subsequent sections.

**Section 2.1 **

- This section discusses very important considerations when it comes to different sequencing technologies. Since sequencing technologies are constantly evolving perhaps it would be relevant to add some future perspectives, e.g. how to deal with emerging outperforming technologies. 

- Paragraph 2: From the sentence starting with “It is important, in this case...”, it is not clear whether the authors mean the bias among different platforms’ outputs or outputs from the same platform.

**Section 2.2 **

- Apart from being in silico generated or obtained from a real-life sample, the data can come from different types of samples. In the case of metagenomics, it would be an important consideration for the evaluation of bioinformatics tools (i.e. a human gut sample or a soil sample would be characterised by a very different complexity).
- Paragraph 7: “The main issues then are: a) there is a need to demonstrate that the experiment met the necessary quality criteria (see section 3.3)” - section 3.3 does not contain any information on the quality criteria.
- Paragraph 11: “Because each approach has advantages and disadvantages, the choice must be carefully considered, according to the purpose of the dataset, which will be discussed in section 4.” - section 4 is missing.

**Section 2.4 **

- This is perhaps the most important section considering the focus of the paper on AMR detection, however, it is very concise and does not provide a good overview of the challenges (e.g. what type of AMR mechanisms to include, which pathogens to consider). These topics are described later in section 3.1 in the example of a particular use case. However, it would be beneficial to outline them in the General considerations section.
- Paragraph 2: “These are specific to the purpose of the dataset (Figure 1) and will be discussed in section 4.1–section 4.3 below” - sections 4.1-4.3 are missing.

**Section 2.5**

- To aid understanding it would be helpful to clarify genomic and phenotypic endpoint, perhaps by adding to the first sentence, e,g, “..a) they can detect the genetic determinants of AMR (genomic endpoint), and in addition b) some can predict the AMR/susceptibility of the bacteria in the original sample (phenotypic endpoint).”

**Section 3.1**

- Paragraph 4: “3. Combinations of (1) and (2) present in at least one of the chosen lists (see cells in Table 2), the sequences are combined and used as the input to simulate the reads using the appropriate tools (see section 3.2).” - maybe the authors meant section 2.3 instead of 3.2.
- Paragraph 6: “The endpoint considered for this benchmark is thus genotypic (see section 3.5),” - section 3.5 is missing.
- Paragraph 10: “When generated, the benchmark should be deployed on a dedicated (and sustainably maintained) platform that includes all the links to the data (see section 3.6) ” - section 3.6 is missing.

**Conclusions**

- The authors mention in the conclusion that they identified two main use cases for this benchmarking resource, each necessitating its own platform: single isolates and mixed samples. This could be expanded upon in the General considerations section to give the reader an understanding of the different challenges and approaches for the two use cases. It is also implied, but not specifically stated, in sections 3.1 and 3.2 that they focus on single isolates. This only becomes clear when reading section 3.3.
- Sections 4.1-4.3 are missing.

Is the topic of the opinion article discussed accurately in the context of the current literature?

Yes

Are arguments sufficiently supported by evidence from the published literature?

Yes

Are all factual statements correct and adequately supported by citations?

Yes

Are the conclusions drawn balanced and justified on the basis of the presented arguments?

Yes

Reviewer Expertise:

AMR, NGS data analysis, bioinformatics

We confirm that we have read this submission and believe that we have an appropriate level of expertise to confirm that it is of an acceptable scientific standard, however we have significant reservations, as outlined above.

## References

[1] : Systematic benchmarking of omics computational tools. *Nature Communications* .2019;10(1) : 10.1038/s41467-019-09406-4 10.1038/s41467-019-09406-4 PMC643716730918265

[2] : Critical Assessment of Metagenome Interpretation-a benchmark of metagenomics software. *Nat Methods* .2017;14(11) : 10.1038/nmeth.4458 1063-1071 10.1038/nmeth.4458 28967888PMC5903868

# Response

**Published:** 2021-09-16

**Authors:** Mauro Petrillo

**Affiliations:** European Commission, Joint Research Centre (JRC), Ispra, Italy

Dear Dr Abramova and Dr Wenne,

 Thanks a lot for your valuable comments and suggestions that you have provided in the report.

 We will address all of them, together with those of other reviewers, in order to provide a fully revised version of the manuscript.

 Best regards,

 Mauro Petrillo, on behalf of the authors.

# Response

**Published:** 2022-03-09

**Authors:** Mauro Petrillo

**Affiliations:** European Commission, Joint Research Centre (JRC), Ispra, Italy

*Replies to:*

*Anna Abramova, Department of Infectious Diseases, University of Gothenburg, Gothenburg, Sweden*

*Marcus Wenne, Department of Infectious Diseases, University of Gothenburg, Gothenburg, Sweden*

*REPLY:* *Thanks for your comments, we have revised the whole manuscript and we hope it is now in line with your expectations.*

*Introduction*

- Since the main focus of the paper is benchmarking, it would be beneficial to provide a short background on the previous benchmarking resources/initiatives in the introduction (e.g. Mangul et al., 20191, Sczyrba et al., 20172, etc).

*REPLY:* *Thanks the suggestion. We added them in the Conclusions section.*

*Section 2*

 Paragraph 2: “In the conclusions of the previous article...”, it is confusing which article the authors refer to since reference 19 (Bellman et al., 2015) provided at the end of the sentence does not contain the cited text.

*REPLY:* *Thanks for spotting this error, which has been corrected.*

*General considerations*

- Several important questions such as which tools to include in the benchmarking, should they be run with optimised or default parameters (e.g. default or customised database), and what performance metrics are to be used for evaluation - could be added to the "General discussion" to clarify and benefit in the understanding of the subsequent sections.

*REPLY:* *Section 3.1 was substantially rewritten to provide more information on the performance-based evaluation. Concretely, both tools and their parameters, should be open to the preferences and requirements of the end users. Certain groups might have a preference for certain tools because it is easier to implement for them, they have developed the tool themselves in-house, it’s freely available as open-source solutions or alternatively commercially available but with full customer support etc. As long as said tools are demonstrated to provide a certain minimum performance, they can be used since the benchmarking should focus on performance rather than enforcing a single pipeline to be used by all (which at any rate, would be unlikely, given the plethora of different sequencing technologies, platforms, and chemistries). The same logic applies to settings, for which certain groups may wish to extensively finetune certain settings but others may prefer to keep them at defaults. With respect to the actual performance metrics to be used, more information was provided in the revised manuscript but we prefer redirecting to reference publications such as Kozyreva et al. (* *https://doi.org/10.1128/JCM.00361-17* *) for a more detailed explanation and example of how these could be implemented, since providing all that information would substantially enlarge the size of this paper.*

*Section 2.1 *

- This section discusses very important considerations when it comes to different sequencing technologies. Since sequencing technologies are constantly evolving perhaps it would be relevant to add some future perspectives, e.g. how to deal with emerging outperforming technologies.

*REPLY:* *A sentence has been added to the end of the section indicating that implementation of new benchmark datasets should be prioritized if new and better technologies emerge.*

- Paragraph 2: From the sentence starting with “It is important, in this case...”, it is not clear whether the authors mean the bias among different platforms’ outputs or outputs from the same platform.

*REPLY:* *Text has been modified to try to clarify that this means bias among the different platforms.*

*Section 2.2 *

- Apart from being in silico generated or obtained from a real-life sample, the data can come from different types of samples. In the case of metagenomics, it would be an important consideration for the evaluation of bioinformatics tools (i.e. a human gut sample or a soil sample would be characterised by a very different complexity).

*REPLY:* *We have addressed this point in section 3, which was revised.*

- Paragraph 7: “The main issues then are: a) there is a need to demonstrate that the experiment met the necessary quality criteria (see section 3.3)” - section 3.3 does not contain any information on the quality criteria.

*REPLY:* *Should be 2.3* *Text has been modified accordingly.*

  

- Paragraph 11: “Because each approach has advantages and disadvantages, the choice must be carefully considered, according to the purpose of the dataset, which will be discussed in section 4.” - section 4 is missing.

*REPLY:* *Should be 3.* *Text has been modified accordingly.*

*Section 2.4 *

- This is perhaps the most important section considering the focus of the paper on AMR detection, however, it is very concise and does not provide a good overview of the challenges (e.g. what type of AMR mechanisms to include, which pathogens to consider). These topics are described later in section 3.1 in the example of a particular use case. However, it would be beneficial to outline them in the General considerations section.

*REPLY:* *The “General considerations” section has been expanded accordingly.*

  

- Paragraph 2: “These are specific to the purpose of the dataset (Figure 1) and will be discussed in section 4.1–section 4.3 below” - sections 4.1-4.3 are missing.

*REPLY:* *Should be 3.1-3.3.* *Text has been modified accordingly.*

*Section 2.5*

- To aid understanding it would be helpful to clarify genomic and phenotypic endpoint, perhaps by adding to the first sentence, e,g, “..a) they can detect the genetic determinants of AMR (genomic endpoint), and in addition b) some can predict the AMR/susceptibility of the bacteria in the original sample (phenotypic endpoint).”

*REPLY:* *Added as suggested.*

*Section 3.1*

- Paragraph 4: “3. Combinations of (1) and (2) present in at least one of the chosen lists (see cells in Table 2), the sequences are combined and used as the input to simulate the reads using the appropriate tools (see section 3.2).” - maybe the authors meant section 2.3 instead of 3.2.

*REPLY:* *Should be 2.3, text has been modified.*

- Paragraph 6: “The endpoint considered for this benchmark is thus genotypic (see section 3.5),” - section 3.5 is missing.

*REPLY:* *Should be 2.5- Text has been modified accordingly*

- Paragraph 10: “When generated, the benchmark should be deployed on a dedicated (and sustainably maintained) platform that includes all the links to the data (see section 3.6) ” - section 3.6 is missing.

*REPLY:* *Should be 2.6. Text has been modified accordingly*

*Conclusions*

- The authors mention in the conclusion that they identified two main use cases for this benchmarking resource, each necessitating its own platform: single isolates and mixed samples. This could be expanded upon in the General considerations section to give the reader an understanding of the different challenges and approaches for the two use cases. It is also implied, but not specifically stated, in sections 3.1 and 3.2 that they focus on single isolates. This only becomes clear when reading section 3.3.

*REPLY:* *A short introduction in section 3 has been added. The “General considerations” section has been expanded accordingly, too.*

  

- Sections 4.1-4.3 are missing.

*REPLY:* *Should be section 3.* *Text has been modified accordingly.*

# Peer review: Reviewer response for version 1

**DOI:** 10.5256/f1000research.42277.r84352

**Published:** 2021-05-21

**Authors:** Rene Hendriksen

**Affiliations:** 1 National Food Institute, Technical University of Denmark, Bygning, Lyngby, Denmark

This manuscript describes a road map how to set up and conduct benchmarking to assess bioinformatics pipelines to detect AMR genes in three levels.

**Overall, comments:**

 The manuscript is well-written but I find it in several paragraphs hard to comprehend the sentences as the authors contradict themselves. This needs to be address as the topic is important but it needs to be all clear.

 I suggest to focus the paper on single isolate genomes rather than but this and metagenomics. It is really two separate technologies and will need different approaches. It is trying to explain all but fails really to in depth address metagenomics.

**Specific comments:**

- Page 4 1 ^st^  paragraph: Describe other epidemiological traits alongside with the timeline and relatedness by merging the paragraphs “….such as virulence, resistance to antibiotics, typing and other adaptive traits… to the same paragraph. I was missing the characterization of AMR genes in the initial lines as this is the focus and title of the paper.
- Page 4 introduction: I miss an explanation about “complex microbial communities”. I know this is metagenomics but the term needs to be introduced.
- Page 4 introduction: “…. antimicrobial resistance (AMR) genetic determinants from NGS data…. This needs to be clarified if this include acquired antimicrobial resistance genes AND/ OR chromosomal point mutations. This is not clear what the approach includes.
-  Page 5 2 ^nd^ bullet: Clarify what is mean by resistome. I suggest to make it clear that this bullet deals with metagenomics.
- Page 5 last paragraph: “Bioinformatics pipelines are thus usually designed to handle the output of a specific platform, often in a certain configuration”. This is true but also the achilles heel of the further description where this is being contradicted.
- Fig 1: I miss the word “concordance to phenotype” as well as curation for scope 1.
- Page 6 2 ^nd^ paragraph: “in practice, a prioritisation exercise should be made based on the capacity building efforts in testing laboratories”. Clarify why this is needed!
- Fig 2: The data could easily be explained in text – omit fig 2 or reference already published similar figures.
- Page 6 4 ^th^ paragraph: I don’t understand the concept. It was earlier explained that “Bioinformatics pipelines are thus usually designed to handle the output of a specific platform, often in a certain configuration” so, why compare the output of a certain pipeline from data generated from different platforms knowing that the result for certain platforms will be biased due to the low comparability of a certain pipeline.
- Page 6 5 ^th^ paragraph: “The FASTQ format is a standard format in this context, which should be used in the benchmark resources; many tools exist to convert the raw data output files into this format in case of different platform outputs (see, for example,39,40) although, it should be noted, different tools may produce different results and this step should be carefully planned.” I find this a source for bring in bias to the benchmarking. I find it hard to see how one can trust the analysis when bringing in variation which might not even be controlled.
- Page 7 1 ^st^, 2 ^nd^, 7 ^th^ paragraph: I did it contradicting that its phrased that “ Although the disadvantage of simulating in silico data is obvious (it is not ‘real’), there are some substantial advantages: it is a lot cheaper than performing sequencing runs, a lot faster, and can be applied to any genome previously sequenced.” and “However, a major drawback is that simulating variation the way nature evolves is very challenging – genetic variation happens in places in the genome where it is hardest to find.” And “although this requires strict annotation of the experiment; c) it will not be possible (besides rare exceptions) to build datasets for the different platforms using the same initial samples.” First of all, its not all that can prepare simulated datasets and secondly, its correct that it will never mimic nature, Thus, I don’t see why this is so heavily recommended.
- Page 9 1 ^st^ paragraph: I miss N50, no of contigs etc.. to be mentioned as QC metrics.
- Page 9 2 ^nd^ paragraph: Not easy to understand.
- Page 9 2.4 1 ^st^ paragraph: “a very pragmatic approach could be the generation of random DNA sequences, to which particular sequences of interest are added (i.e. fragments of AMR genes). However, there is sufficient evidence that the genomic background of the bacteria (i.e. the “non-AMR related” sequences) can have a profound influence on the performance of the pipelines”. Contradiction – see Page 7 1 ^st^, 2 ^nd^ , 7 ^th^ paragraph.
- Page 9 6 ^th^ paragraph: define “phenotypic endpoint”.
- Page 9 6 ^th^ paragraph: “Studies that evaluated AMR genotype to phenotype relationships have indicated that despite generally high correspondence, this can vary greatly between pathogens / case studies, and even for different antimicrobial agents within the same species 57,58.” I need to be further elaborated – what do one trust the phenotypic data or detected genes.
- Page 9 intro to bullets: define “genomic endpoint”.
- Page 9 section 2.6: Agree that metadata is needed but it needs to be explained that its not needed for the benchmarking itself but for others to use the dataset for future exercises.
- Page 10 1 ^st^ paragraph of section 3.1: explain what is meant by “agreed minimum standards”- what performance metrics.
- Page 10 3 ^rd^ paragraph of section 3.1: I find it contradicting to Page 7 1 ^st^, 2 ^nd^, 7 ^th^ paragraph.
- Page 10: WHO CIA has been updated – provide ref.
- Page 10: The decision has been updated in 2021 – provide ref.
- Page 13: Tabel 3 greatly lack a million PT/ EQA reports from EURLs e.g. https://www.eurl-ar.eu/reports.aspx https://antimicrobialresistance.dk/eqas.aspx
- Page 14 section 3.3: I would omit this part as it add more confusion to bring in also metagenomics to the concept – a completely different approach with complex samples. Past studies has also show that benchmarking metagenomics is not a trivial discipline.

Is the topic of the opinion article discussed accurately in the context of the current literature?

Partly

Are arguments sufficiently supported by evidence from the published literature?

No

Are all factual statements correct and adequately supported by citations?

Partly

Are the conclusions drawn balanced and justified on the basis of the presented arguments?

Partly

Reviewer Expertise:

Microbiologist focusing on NGS and EQA/ benchmarking

I confirm that I have read this submission and believe that I have an appropriate level of expertise to confirm that it is of an acceptable scientific standard, however I have significant reservations, as outlined above.

# Response

**Published:** 2021-09-16

**Authors:** Mauro Petrillo

**Affiliations:** European Commission, Joint Research Centre (JRC), Ispra, Italy

Dear Dr Hendriksen,

 Thanks a lot for your valuable comments and suggestions that you have provided in the report.

 We will address all of them, together with those of other reviewers, in order to provide a fully revised version of the manuscript.

 Best regards,

 Mauro Petrillo, on behalf of the authors.

# Response

**Published:** 2022-03-09

**Authors:** Mauro Petrillo

**Affiliations:** European Commission, Joint Research Centre (JRC), Ispra, Italy

*Replies to Rene Hendriksen, National Food Institute, Technical University of Denmark, Bygning, Lyngby, Denmark* *.*

***Overall, comments***

 The manuscript is well-written but I find it in several paragraphs hard to comprehend the sentences as the authors contradict themselves. This needs to be address as the topic is important but it needs to be all clear.

 I suggest to focus the paper on single isolate genomes rather than but this and metagenomics. It is really two separate technologies and will need different approaches. It is trying to explain all but fails really to in depth address metagenomics.

*REPLY:* *Thanks for your comments, we have revised the whole manuscript and we hope it is now in line with your expectations.*

***Specific comments***

- Page 4 1st  paragraph: Describe other epidemiological traits alongside with the timeline and relatedness by merging the paragraphs “….such as virulence, resistance to antibiotics, typing and other adaptive traits… to the same paragraph. I was missing the characterization of AMR genes in the initial lines as this is the focus and title of the paper.

*REPLY:* *Paragraphs 1 and 2 have been merged, and reference to the use of this data for inferring AMR has been moved to early in paragraph 1 as suggested.*

- Page 4 introduction: I miss an explanation about “complex microbial communities”. I know this is metagenomics but the term needs to be introduced.

*REPLY:* *As requested, bullets on this page have been rewritten to introduce the “metagenome” terminology*

- Page 4 introduction: “…. antimicrobial resistance (AMR) genetic determinants from NGS data…. This needs to be clarified if this include acquired antimicrobial resistance genes AND/ OR chromosomal point mutations. This is not clear what the approach includes.

*REPLY:* *The bullets on page 4 have been modified to indicate that phenotype prediction relies on detection of both acquired ARGs and point mutations. We felt that this was a better place to define the types of genetic determinants associated with AMR*

  

- Page 5 2nd bullet: Clarify what is mean by resistome. I suggest to make it clear that this bullet deals with metagenomics.

*REPLY:* *This bullet has been rewritten to address clarity*.

- Page 5 last paragraph: “Bioinformatics pipelines are thus usually designed to handle the output of a specific platform, often in a certain configuration”. This is true but also the achilles heel of the further description where this is being contradicted.

*REPLY:* *We have rewritten section 3.1 to make clearer that validation of different pipelines should be performance-based, i.e. focus on their performance rather than enforcing a single pipeline to be adopted by the community. Different sequencing technologies exist, but also for the same technology there exist differences in instruments and chemistries that potentially require specifically adopted pipelines to accommodate specific configurations. By building community benchmarking datasets for which the ground truth is well-established for those configurations, prioritizing dominant technologies, it becomes possible that different pipelines are used by different groups (even when both groups otherwise use exactly the same configuration), as long as those pipelines meet minimum agreed upon acceptance values for their performance.*

- Fig 1: I miss the word “concordance to phenotype” as well as curation for scope 1.

*REPLY:* *Sorry, we did not understand this point. Do you mean these concepts should be added to Figure 1? Or that what is reported in the figure 1 is inconsistent with what mentioned in the text?*

- Page 6 2nd paragraph: “in practice, a prioritisation exercise should be made based on the capacity building efforts in testing laboratories”. Clarify why this is needed!

*REPLY:* *This has been reworded to indicate that it may be necessary if resource limitations are an issue. *

- Fig 2: The data could easily be explained in text – omit fig 2 or reference already published similar figures.

*REPLY:* *We added additional text to justify the presence of Figure 2.*

- Page 6 4th paragraph: I don’t understand the concept. It was earlier explained that “Bioinformatics pipelines are thus usually designed to handle the output of a specific platform, often in a certain configuration” so, why compare the output of a certain pipeline from data generated from different platforms knowing that the result for certain platforms will be biased due to the low comparability of a certain pipeline.

*REPLY:* *We provide here a clarification. Even though pipelines can be drastically different, e.g. made for different sequencing technologies or use completely different algorithmic approaches for a certain configuration (e.g. assembly-based or read-mapping based strategies), their final aim in the context of this manuscript considers the correct detection and identification of AMR determinants (whether SNPs, genes or more complex features), as quantified by validating their performance. Consequently, it is not the output of those pipelines themselves that will be directly compared, but rather the ability of said pipelines to correctly detect and identify AMR determinants. Whatever sequencing technology or bioinformatics methodology is applied, if a pipeline designed to detect AMR genes cannot properly detect and identify those genes, then said pipeline cannot be considered validated for AMR gene detection in a clinical or regulatory application. Contrarily, if said pipeline can correctly detect and identify those genes, then it could be applied in such contexts.*

- Page 6 5th paragraph: “The FASTQ format is a standard format in this context, which should be used in the benchmark resources; many tools exist to convert the raw data output files into this format in case of different platform outputs (see, for example,39,40) although, it should be noted, different tools may produce different results and this step should be carefully planned.” I find this a source for bring in bias to the benchmarking. I find it hard to see how one can trust the analysis when bringing in variation which might not even be controlled.

*REPLY:* *This is exactly what the generation of community benchmark datasets circumvents by providing reference datasets for which the ground truth is well-established. By starting from such samples, whether using reference genomes with known AMR determinants for which reads are simulated with error profiles modelled to mimic specific sequencing technologies, or alternatively using real samples where certain AMR determinants have been shown to be present/absent with traditional methods (e.g. PCR and/or Sanger sequencing) subjected to sequencing by specific sequencing technologies, the resulting datasets can be analyzed with pipelines for which it is known which AMR determinants should be detected. If such an approach uncovers that certain variation and biases specific to certain sequencing technologies and/or configurations cannot be controlled for bioinformatically and negatively affect pipeline performance leading to missed detection (or alternatively false positive detections), said pipeline cannot be considered validated for AMR characterization in a clinical or regulatory application. We hope this clarifies.*

- Page 7 1st, 2nd, 7th paragraph: I did it contradicting that its phrased that “ Although the disadvantage of simulating in silico data is obvious (it is not ‘real’), there are some substantial advantages: it is a lot cheaper than performing sequencing runs, a lot faster, and can be applied to any genome previously sequenced.” and “However, a major drawback is that simulating variation the way nature evolves is very challenging – genetic variation happens in places in the genome where it is hardest to find.” And “although this requires strict annotation of the experiment; c) it will not be possible (besides rare exceptions) to build datasets for the different platforms using the same initial samples.” First of all, its not all that can prepare simulated datasets and secondly, its correct that it will never mimic nature, Thus, I don’t see why this is so heavily recommended.

*REPLY:* *We have rewritten section 3.1 to take the suggestion of the reviewer into account by rendering the second approach for generating benchmark data, i.e. sequencing of real samples on the condition that their AMR determinants are well-established by other approaches (such as conventional PCR and/or Sanger sequencing), more prominent.*

- Page 9 1st paragraph: I miss N50, no of contigs etc.. to be mentioned as QC metrics.

*REPLY:* *We have now included quality of the assembly as an example of an important QC metric.*

- Page 9 2nd paragraph: Not easy to understand.

*REPLY:* *This paragraph has been rewritten for clarity.*

- Page 9 2.4 1st paragraph: “a very pragmatic approach could be the generation of random DNA sequences, to which particular sequences of interest are added (i.e. fragments of AMR genes). However, there is sufficient evidence that the genomic background of the bacteria (i.e. the “non-AMR related” sequences) can have a profound influence on the performance of the pipelines”. Contradiction – see Page 7 1st, 2nd , 7th paragraph.

*REPLY:* *Thanks for spotting this contradiction. It has been clarified by rephrasing.*

-  page 9 6th paragraph: define “phenotypic endpoint”.

*REPLY:* *This has been defined in the first paragraph of section 2.5*

- Page 9 6th paragraph: “Studies that evaluated AMR genotype to phenotype relationships have indicated that despite generally high correspondence, this can vary greatly between pathogens / case studies, and even for different antimicrobial agents within the same species 57,58.” I need to be further elaborated – what do one trust the phenotypic data or detected genes.

*REPLY:* *The subsequent paragraphs explain that focusing on a genomic endpoint has advantages. The name of the section has been change, as its aim is not to assess what is better between the two described endpoints.*

- Page 9 intro to bullets: define “genomic endpoint”.

*REPLY:* *This has been defined in the first paragraph of section 2.5*

- Page 9 section 2.6: Agree that metadata is needed but it needs to be explained that its not needed for the benchmarking itself but for others to use the dataset for future exercises.

*REPLY:* *Added a sentence to clarify this. *

- Page 10 1st paragraph of section 3.1: explain what is meant by “agreed minimum standards”- what performance metrics.

*REPLY:* *We have substantially revised section 3.1 to render the “agreed minimum standards” clearer.*

- Page 10 3rd paragraph of section 3.1: I find it contradicting to Page 7 1st, 2nd, 7th paragraph.
- Page 10: WHO CIA has been updated – provide ref.
*REPLY: the WHO* *web  page* *states the a new update will be issued in 2022. WHO release: 2020 annual review of the clinical and preclinical antibacterial pipelines evaluates the potential of antibacterial candidates (https://www.who.int/publications/i/item/9789240021303)*
- Page 10: The decision has been updated in 2021 – provide ref.
*REPLY: changed according to request.*
- Page 13: Tabel 3 greatly lack a million PT/ EQA reports from EURLs e.g. https://www.eurl-ar.eu/reports.aspx https://antimicrobialresistance.dk/eqas.aspx

*REPLY:* *We agree that a lot of PT/ EQA reports from EURLs exist. However, Table 3 is proposed as “a non-exhaustive list of recent references to be used as a starting point” and, among them, reference 85 is a report from the EURL-AR. Anyway, we modified the text to highlight the relevance of these reports.*

 Page 14 section 3.3: I would omit this part as it add more confusion to bring in also metagenomics to the concept – a completely different approach with complex samples. Past studies has also show that benchmarking metagenomics is not a trivial discipline.

*REPLY:* *We did not omit this part as we believe it is linked to the resistome concept that we addressed, thanks to your suggestions.*
